# Type I IFN Signaling Is Dispensable during Secondary Viral Infection

**DOI:** 10.1371/journal.ppat.1005861

**Published:** 2016-08-31

**Authors:** Martin P. Hosking, Claudia T. Flynn, J. Lindsay Whitton

**Affiliations:** Department of Immunology and Microbial Science, The Scripps Research Institute, La Jolla, California, United States of America; Emory University, UNITED STATES

## Abstract

Innate immune responses in general, and type I interferons (T1IFNs) in particular, play an important and often essential role during primary viral infections, by directly combatting the virus and by maximizing the primary adaptive immune response. Several studies have suggested that T1IFNs also contribute very substantially to the secondary (recall) response; they are thought (i) to be required to drive the early attrition of memory T cells, (ii) to support the subsequent expansion of surviving virus-specific memory cells, and (iii) to assist in the suppression and clearance of the infectious agent. However, many of these observations were predicated upon models in which T1IFN signaling was interrupted prior to a primary immune response, raising the possibility that the resulting memory cells might be intrinsically abnormal. We have directly addressed this by using an inducible-Cre model system in which the host remains genetically-intact during the primary response to infection, and in which T1IFN signaling can be effectively ablated prior to secondary viral challenge. We report that, in stark contrast to primary infection, T1IFN signaling is not required during the recall response. IFNαβR-deficient memory CD8^+^ and CD4^+^ memory T cells undergo attrition and expansion with kinetics that are indistinguishable from those of receptor-sufficient cells. Moreover, even in the absence of functional T1IFN signaling, the host’s immune capacity to rapidly suppress, and then to eradicate, a secondary infection remains intact. Thus, this study shows that T1IFN signaling is dispensable during the recall response to a virus infection. Moreover, two broader implications may be drawn. First, a T cell’s requirement for a cytokine is highly dependent on the cell’s maturation / differentiation status. Consequently, second, these data underscore the importance of evaluating a gene’s impact by modulating its expression or function in a temporally-controllable manner.

## Author Summary

Type I interferons (T1IFNs) are key pleiotropic anti-viral cytokines, necessary to effectively promote and coordinate immune responses and control a number of primary viral infections. In their absence, the expansion and later memory formation of anti-viral T cells is severely curtailed. T1IFNs are also thought to perform a similar role during a recall response, although whether this signaling axis is truly required is obscured by the fact that initial immune responses, and by extension any memory cells subsequently generated, in the absence of these cytokines are potentially defective. Here we have employed an inducible-deletion model, wherein normal responses to primary infections proceed unhindered, and only after the successful establishment of long-term memory T cells is the common receptor for T1IFNs, IFNαβR, deleted. We demonstrate that, unlike during primary infection, both the recall immune response and control of virus is independent of functional T1IFN signaling. Importantly, our results highlight a need to re-evaluate the critical and requisite determinants of a successful recall response in a temporally-controlled manner.

## Introduction

The recognition of pathogen associated molecular patterns (PAMPs), including single stranded endosomal RNA, by pattern recognition receptors such as TLR7 within virally infected cells and/or specialized secreting cells (e.g. plasmacytoid dendritic cells) induces the production of pro-inflammatory cytokines, including the type I interferons (T1IFNs), IFNα and IFNβ [reviewed, 1]. Signaling through their common receptor IFNαβR to activate the Jak/Stat intracellular signaling cascade [reviewed, 2], type I IFNs occupy an often critical lynchpin of productive anti-viral responses: (i) inducing a local anti-viral state through the upregulation of interferon stimulatory genes (ISGs) capable of limiting the replication of a broad range of viruses within infected and neighboring cells [3] and (ii) activating and, in general, promoting both innate and adaptive arms of the immune system [reviewed, 4,5]. The inability of IFNαβR-deficient mice to control a myriad of viral infections, including lymphocytic choriomeningitis virus (LCMV) [6–8], highlights the importance of an intact type I IFN signaling system *in vivo*.

T1IFNs directly promote dendritic cell maturation, antigen processing, and costimulation expression [9–12], leading to greater and more potent *in* vitro [10,13] and *in vivo* [12,14] T cell expansion and effector function. In addition, during certain viral infections T1IFN can provide a potent “signal 3,” aiding in the activation of CD8^+^ T cells, even in the absence of CD4^+^ T cell help [15]. Further, T1IFN also directly regulates T lymphocyte expansion and differentiation during viral infection, as antigen-specific CD8^+^ T cells lacking T1IFN receptor fail to appropriately expand *in vivo* during certain primary viral infections, including LCMV [16–19], in part, due to their inability to upregulate NK cell regulatory molecules (e.g. MHC Class I, Qa-1, NCR1) and escape NK cell dependent lysis [20,21], thereby greatly limiting (up to 100-fold) the number of memory T cells generated. Notably, during other primary viral or intracellular bacterial infections, including vaccinia, VSV, or Listeria monocytogenes [18,22], the requirement for functional IFNα/β signaling upon CD8^+^ T cells is not nearly as severe, although T1IFN still, broadly speaking, promotes CD8^+^ T cell expansion. Whether memory CD8^+^ T cell function *in vivo* is similarly predicated upon T1IFN signaling is not clear. Resting IFNαβR-deficient memory CD8^+^ T cells are capable of degranulation and cytokine production following *in vitro* restimulation [19,22]. Secondary expansion and *in vivo* effector function of IFNαβR-deficient memory CD8^+^ T cells has been shown to be curtailed during secondary infection with recombinant *Vaccinia* or LCMV [20,22], whereas an additional study by Oexnius and colleagues detailed no similar deficits in secondary expansion during recombinant Vaccinia virus infection [19].

T1IFN can also prove to be detrimental towards the host or the immune system in general. For example, IFNα/β potentiates leukopenia [23,24], and, more specifically, is a mediator of CD8^+^ T cell attrition [25,26] during viral infection. Pre-exposure to IFN limits CD8^+^ T cell expansion and proliferation [27], and prolonged and aberrant IFN expression has been implicated in the dysregulated anti-viral responses observed during chronic, persistent viral infection [28,29]. Finally, T1IFN is also key to the development of immunopathology following influenza and SARS-CoV infection [30,31] and during models of viral-induced hemorrhage [32,33].

Since T1IFN exerts disparate and pleiotropic influences on both the immune system and most somatic cells, conventional methods including germline and/or conditional knockout models that would limit requisite IFN signaling during primary infection are inappropriate means to assess whether memory responses to virus are similarly dependent upon IFNα/β-signaling, since it would be impossible to separate whether an observed deficiency in secondary immune function was the consequence of an inherent and preexisting deficit or a true reflection for concurrent IFN signaling during secondary viral infection. Here we have utilized an inducible-Cre system to temporally delete IFNαβR from both total immune and parenchymal cells prior to secondary viral infection. Importantly, prior to gene deletion, maturation of naïve T cells, and their responses during and following primary viral infection, are allowed to proceed unhindered. Thus we are able to accurately, and without bias, simultaneously evaluate whether T1IFN influences immune responses and/or viral control during secondary viral infection.

We demonstrate that immediately following secondary viral infection both type I and II IFNs are transiently produced, manifesting both type I and II IFN inducible gene expression within the spleen. Surprisingly, inducible, tamoxifen-mediated deletion of T1IFN receptor signaling during secondary viral infection did not affect the attrition, expansion, quality, or reestablishment of virus-specific memory CD8^+^ T cells. Furthermore, viral control was not abrogated in either the spleen or the liver, demonstrating that a functional T1IFN system is not required for either secondary immune responses or containment of viral replication.

## Results

### Functional type I and II interferons are expressed in response to secondary viral infection

**Fig 1:**
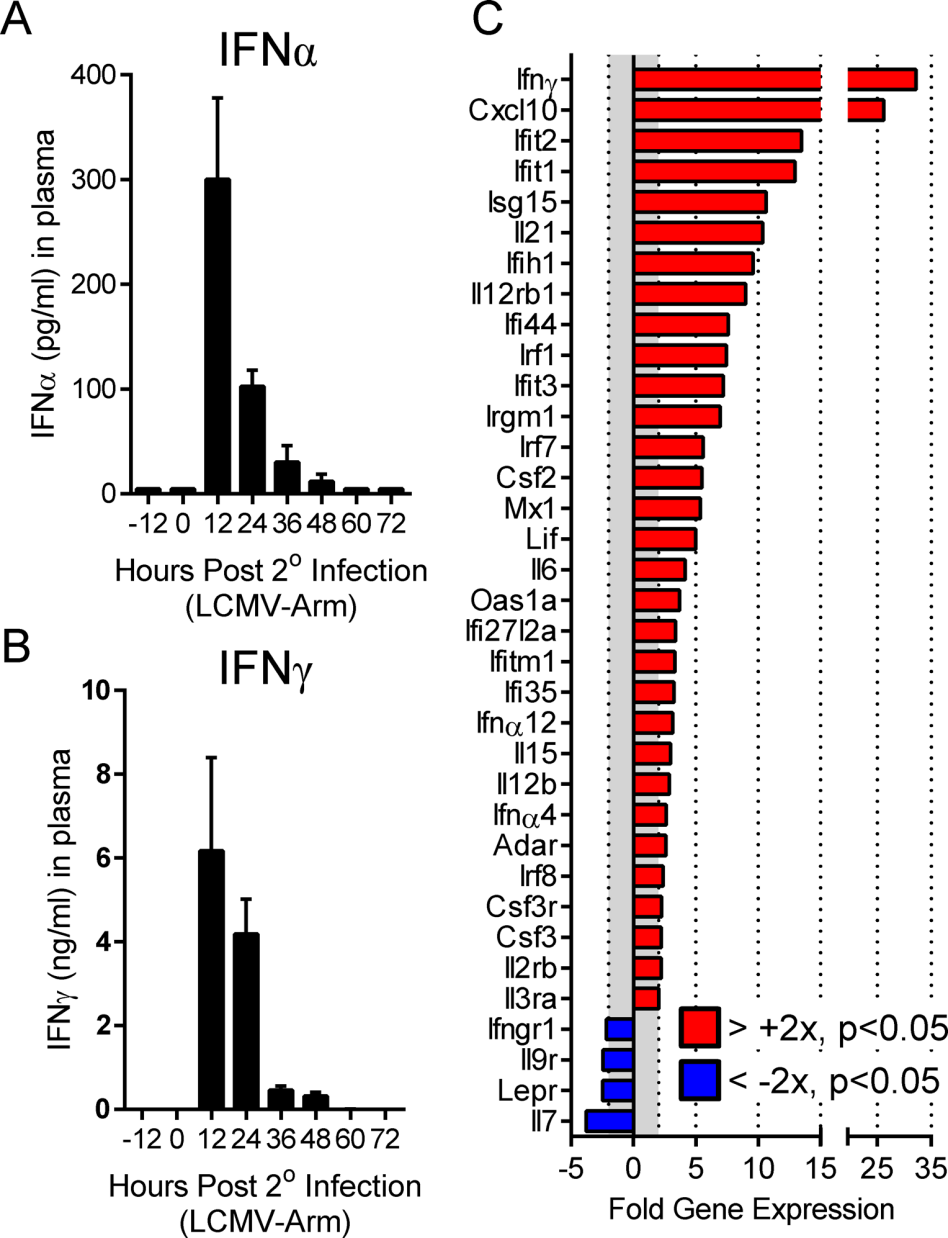
Functional type I and II interferons are expressed in response to secondary viral infection. LCMV-immune mice were rechallenged with 2x10^6^ PFU LCMV-Arm and (A) IFNα and (B) IFNγ levels in the plasma were assessed via ELISA or multiplex, respectively (n = 5 per time point). (C) Type I and type II ISG expression was quantified by PCR array in the spleens of LCMV-immune mice at 12 hours p.i., and the data were normalized to equivalent analyses of sham infected mice (sham n = 6, 12 hrs n = 4).

We evaluated the production, and biological activities, of interferons in the hours immediately following secondary viral infection. Long-term LCMV immune mice (>6 weeks post primary LCMV-Arm) were rechallenged with LCMV, and plasma was collected prior to, and at the indicated times following, the secondary infection; IFNα and IFNγ levels were assessed via ELISA or multiplex assay. Within 12 hours of secondary viral infection, both IFNα and IFNγ became readily detectable within the plasma of infected mice. Thereafter, the expression of both of these cytokines waned dramatically, and within 48–60 hours of infection IFNγ and IFNα were undetectable within the plasma (Fig 1A and 1B). We were unable to detect any IFNβ throughout the course of secondary viral infection. We next determined whether these transient pulses of type I & II IFNs triggered the expression of interferon-inducible genes. Spleens were taken from sham infected LCMV-immune mice, and from LCMV-infected mice at 12 hours p.i., the peak of peripheral IFNα and IFNγ expression. RNA was extracted and subjected to PCR array analysis. Both type I (e.g. MX1, IFIT1-3, Oas1a, & IRF7) and type II (e.g. CXCL10 & IRF1) interferon-inducible genes were significantly upregulated within 12 hours of secondary LCMV infection (Fig 1C). Therefore, during secondary viral infection, type I and II interferons are rapidly but transiently induced, and upregulate the expression of their target genes.

### Tamoxifen-treated naïve Cre^+^IFNαβR^f/f^ mount primary responses that are similar to those reported for conventional IFNαβR knockout animals

**Fig 2:**
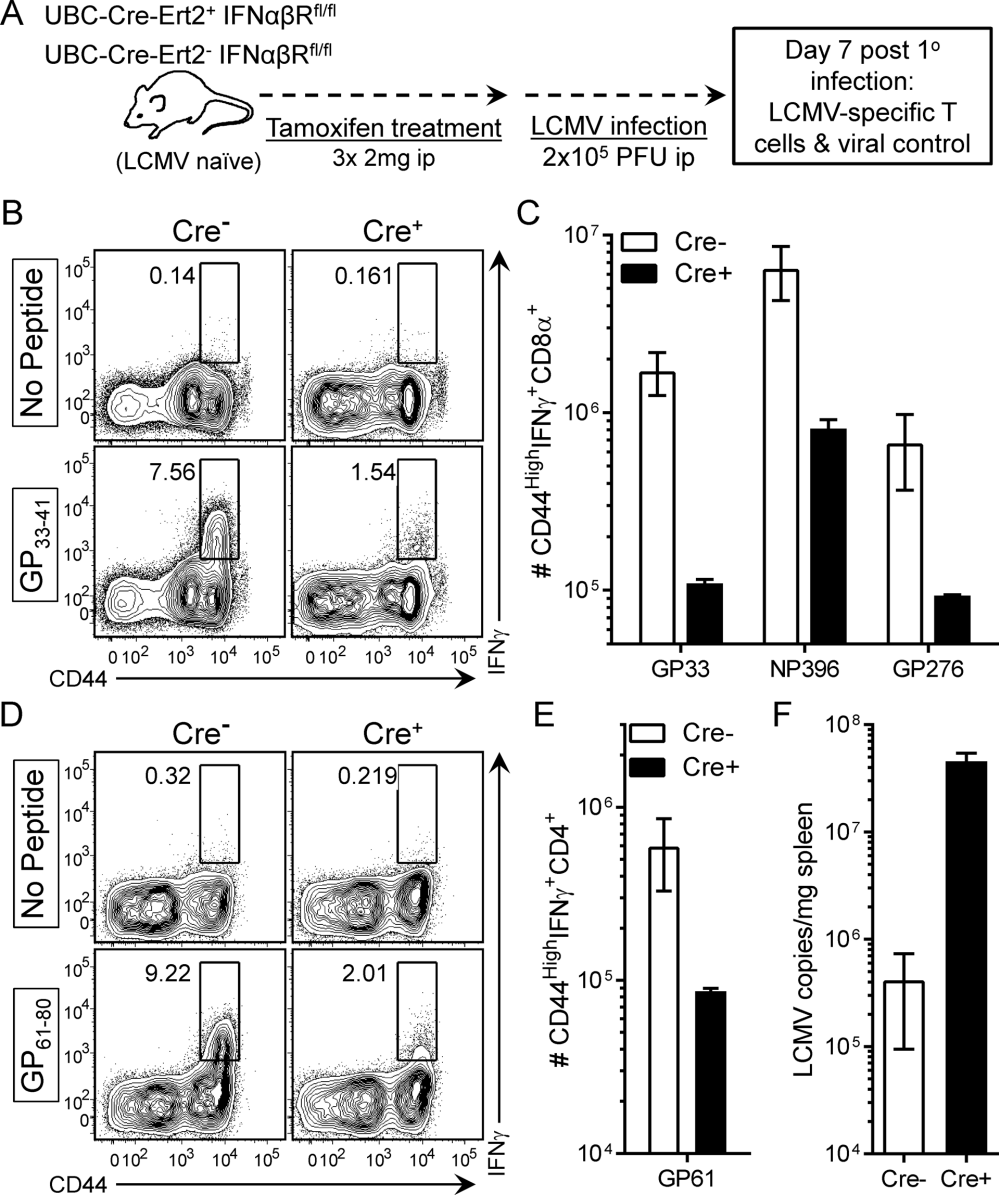
Tamoxifen-treated naïve Cre^+^IFNαβR^f/f^ mount primary responses that are similar to those reported for conventional IFNαβR knockout animals. (A) LCMV naive UBC-Cre-ERT2^+^IFNαβR^fl/fl^ and UBC-Cre-ERT2^-^IFNαβR^fl/fl^ mice were injected with tamoxifen and ~9 weeks later were given a primary infection with LCMV. Seven days p.i., virus specific CD8^+^ (B & C) and CD4^+^ (D & E) T cells were identified and quantified via standard intracellular staining. For each cell type, representative contour plots from individual mice are shown (B & D), followed by cumulative data from all animals (C & E). (F) LCMV vRNA was quantified within the spleens of Cre^+^ and Cre^-^ mice (n = 2).

T1IFN signaling is required to control many primary viral infections *in vivo* [6,7], and the absence of IFNαβR from virus-specific T cells compromises their *in vivo* expansion; this is true for both naive and memory T cells during a number of viral infections [16,17,20,21]. However, the reported defects in memory cell expansion could have resulted from their having developed (i) from receptor-deficient naïve / primary cells and (ii) in an environment lacking T1IFN signaling. To better determine the impact of T1IFN signaling upon recall responses, we developed a model in which IFNαβR ablation could be induced *in vivo* at any time selected by the investigator. Using this approach, the mice are genetically intact prior to, and during, primary infection, ensuring that the resulting memory T cells are developmentally normal; then, IFNαβR ablation can be induced prior to secondary challenge. To this end, UBC-Cre-ERT2 mice, expressing a tamoxifen sensitive variant of cre recombinase under the control of the human ubiquitin C promoter, were bred with IFNαβR^fl/fl^ mice, generating UBC-Cre-ERT2^+^IFNαβR^fl/fl^ and control littermate UBC-Cre-ERT2^-^IFNαβR^fl/fl^ mice. We considered it important to first test the validity of the model, and did so by evaluating the outcome of primary virus infection in naïve mice that had been treated with tamoxifen. In Cre^+^ recipients, tamoxifen exposure should ablate IFNαβR expression, and these mice should recapitulate the published findings with conventional germline IFNαβR knockout mice [6,7,16,17,20,21], which are unable to control primary viral infection, and in which the expansion of virus-specific T cells is severely compromised. Therefore (Fig 2A), naïve UBC-Cre-ERT2^+^IFNαβR^fl/fl^ mice, and their Cre^-^ littermates, were treated with tamoxifen and, several weeks later, were challenged with LCMV-Arm. Seven days p.i., LCMV-specific T cells were enumerated by standard intracellular cytokine staining. UBC-Cre-ERT2^+^ mice were found to have markedly-diminished CD8^+^ T cell responses compared to their tamoxifen-treated Cre^-^ counterparts. Representative responses to the viral GP_33_ epitope in individual Cre^-^ and Cre^+^ mice are shown, demonstrating a marked reduction in the proportion of CD8^+^ T cells that have responded to this epitope (Fig 2B), and cumulative data for this, and two other, LCMV CD8^+^ T cell epitopes revealed >10-fold lower numbers of cells in Cre^+^ animals (Fig 2C). Similar data were observed for GP_61-80_ specific CD4^+^ T cells (Fig 2D and 2E). Finally, these reductions in T cell responses were accompanied by greatly elevated (~100 fold) levels of viral RNA in the spleens of tamoxifen-pretreated Cre^+^ mice, when compared to drug-treated Cre^-^ littermates (Fig 2F).

### Widespread and efficient deletion of IFNαβR in vivo following tamoxifen activation of Cre-ERT2

**Fig 3:**
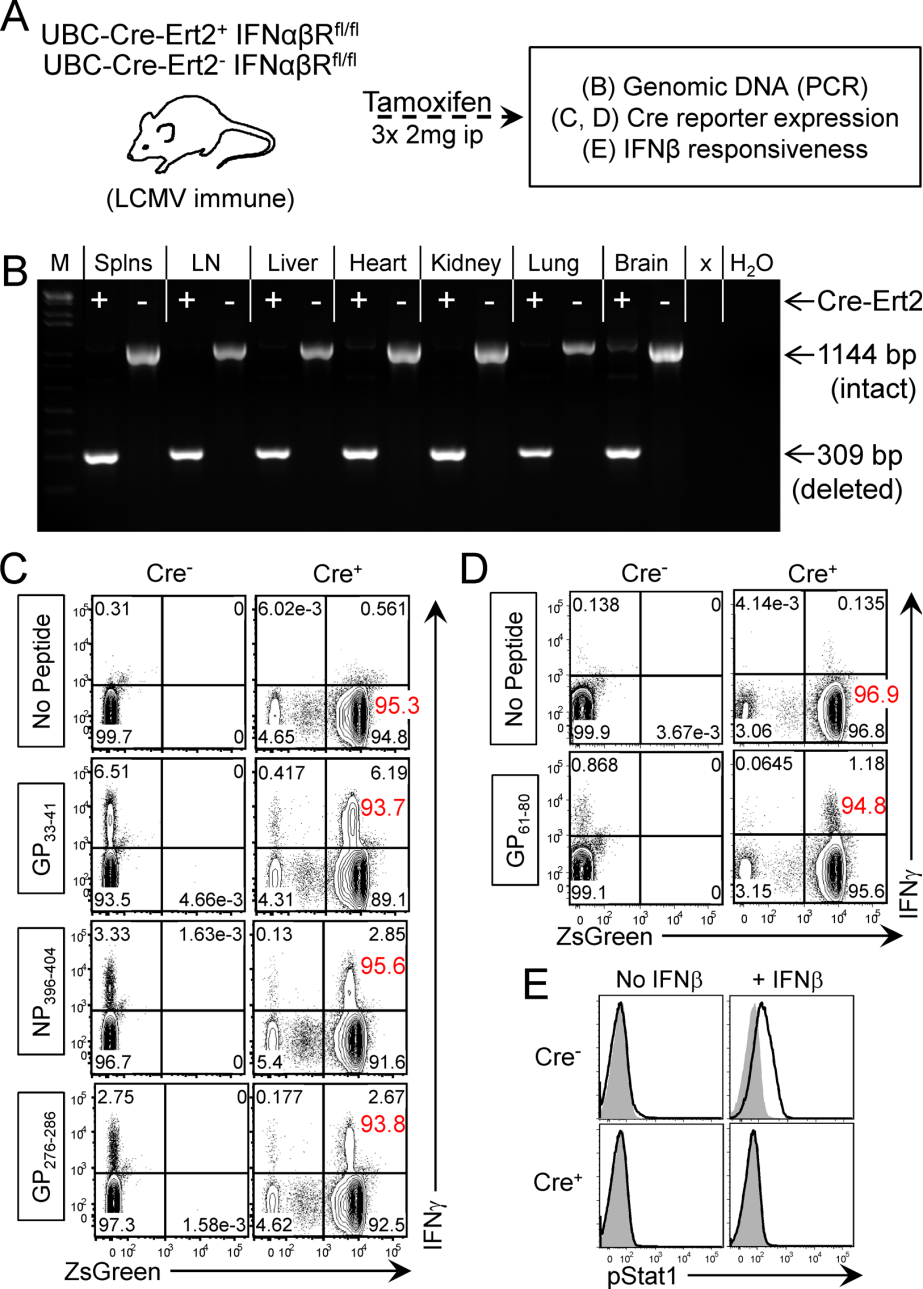
Widespread and efficient deletion of IFNαβR in vivo following tamoxifen activation of Cre-ERT2. (A) LCMV immune UBC-Cre-ERT2^+^IFNαβR^fl/fl^ and UBC-Cre-ERT2^-^IFNαβR^fl/fl^ mice were injected with tamoxifen, and the indicated analyses were carried out. (B) The efficiency of deletion at the DNA level was assessed by PCR analysis of genomic DNA extracted from the indicated tissues; M = markers; Splns = splenocytes; LN = lymph nodes; x = empty lane. 1144bp = floxed allele, 309bp = deleted allele. (C & D) The efficiency of Cre activation in T cells of LCMV-immune mice was determined. Cre^+^ or Cre^-^ IFNαβR^f/f^zsGreen^+/wt^ mice were injected with tamoxifen and, two weeks later, splenocytes were harvested and incubated with each of the four indicated peptides, and virus-specific T cells were identified using standard intracellular cytokine staining for IFNγ. zsGreen expression also was evaluated. The red numbers indicate zsGreen^+^ cells as a percentage of all CD8^+^ T cells (No Peptide groups) or of virus-specific (i.e, IFNγ^+^) cells (peptide-stimulated groups). (C) Gated on CD8^+^ T cells and (D) gated on CD4^+^ T cells. (E) The efficiency with which Cre activation resulted in ablation of IFNαβR biological function was determined, by measuring Stat 1 phosphorylation after *in vitro* incubation with IFNβ. ZsGreen+CD8^+^ T cells from Cre^+^ mice were compared to CD8^+^ T cells from Cre^-^ animals. Grey histograms = isotype control antibody. Mouse numbers: C & D, n = 6–7; E, n = 4.

The above data indicate that tamoxifen-driven IFNαβR deletion is sufficiently effective to functionally recapitulate the impact of a complete genetic knockout during primary viral infection. Thus, we began our evaluation of drug-induced IFNαβR deletion in long-term immune mice. As indicated in Fig 3A, genetically intact Cre^+^ and Cre^-^ IFNαβR^f/f^ mice were infected with LCMV, and were allowed to develop a normal T cell memory pool; then, these long-term LCMV-immune animals were injected with tamoxifen. Several weeks later we assessed the overall *in vivo* efficiency of tamoxifen-induced deletion of IFNαβR, by various criteria. First, genomic DNA was isolated from purified splenocytes, lymph nodes, liver, heart, kidney, lung, and brain. The status of the IFNαβR^f/f^ locus was assessed using PCR primers that flank the loxP sites and, in all of the tissues analyzed, near-complete (brain) or complete (other tissues) deletion of IFNαβR was observed (Fig 3B). Next we determined the efficacy of tamoxifen-mediated Cre activation in T lymphocytes. Cre^+^ and Cre^-^ IFNαβR^f/f^zsGreen^+/wt^ mice, generated as described in materials and methods, were infected with LCMV and allowed to develop into long-term immune mice. Tamoxifen was administered and, two weeks later, CD4^+^ and CD8^+^ T cells were evaluated for zsGreen expression. In Cre^-^ mice, as expected, CD8^+^ memory T cells specific for each of the three indicated epitopes were identified, and the cells did not express zsGreen (Fig 3C, left column). In contrast, in Cre^+^ animals (right column) >90% of CD8^+^ T cells expressed zsGreen, demonstrating that Cre activity is widespread in that cell population (the percentage of zsGreen^+^ cells in each population is shown as a red numeral). Moreover, the ability of tamoxifen to drive Cre enzymatic activity on cellular DNA was not affected by the immunological status of the T cell. The proportion of reporter-expressing cells among all CD8^+^ T cells in the long-term immune Cre^+^ mice (“no peptide”, which will include CD8^+^ T cells that are not LCMV-specific) was ~95.3% [94.8/(4.65 + 94.8)], and a very similar proportion of epitope-specific CD8^+^ T cells were reporter-positive: e.g., 93.7% of GP_33_-responsive (IFNγ^+^) cells [6.19/(0.417 + 6.19)] expressed zsGreen. CD4^+^ T cells presented a similar picture (Fig 3D); in Cre^+^ mice, the vast majority of cells expressed zsGreen, in both total cells (96.9%), and in cells specific for the LCMV GP_61-80_ MHC class II epitope (94.8%). Finally, and most importantly, we determined whether Cre activity, and deletion at the level of genomic DNA, resulted in the functional ablation of T1IFN signaling. Splenocytes were isolated, and stimulated *in vitro* with rIFNβ. As shown in Fig 3E, almost all CD8^+^ T cells from tamoxifen-treated Cre^-^ mice responded to IFNβ stimulation, as judged by increased levels of phosphorylated Stat1. In contrast, zsGreen-expressing CD8^+^ T cells from Cre+ mice failed to upregulate phospho-Stat1 following IFNβ exposure. In summary, tamoxifen treatment of UBC-Cre-ERT2+ mice results in the widespread deletion of IFNαβR, both within peripheral organs and LCMV-specific memory T cells. Moreover, the Cre-mediated removal of IFNαβR quickly renders the memory T cells incapable of responding to T1IFN.

### IFNαβR deletion prior to secondary viral infection does not limit LCMV-specific T cell attrition or the expression of MHC Class I & Qa-1 in vivo

**Fig 4:**
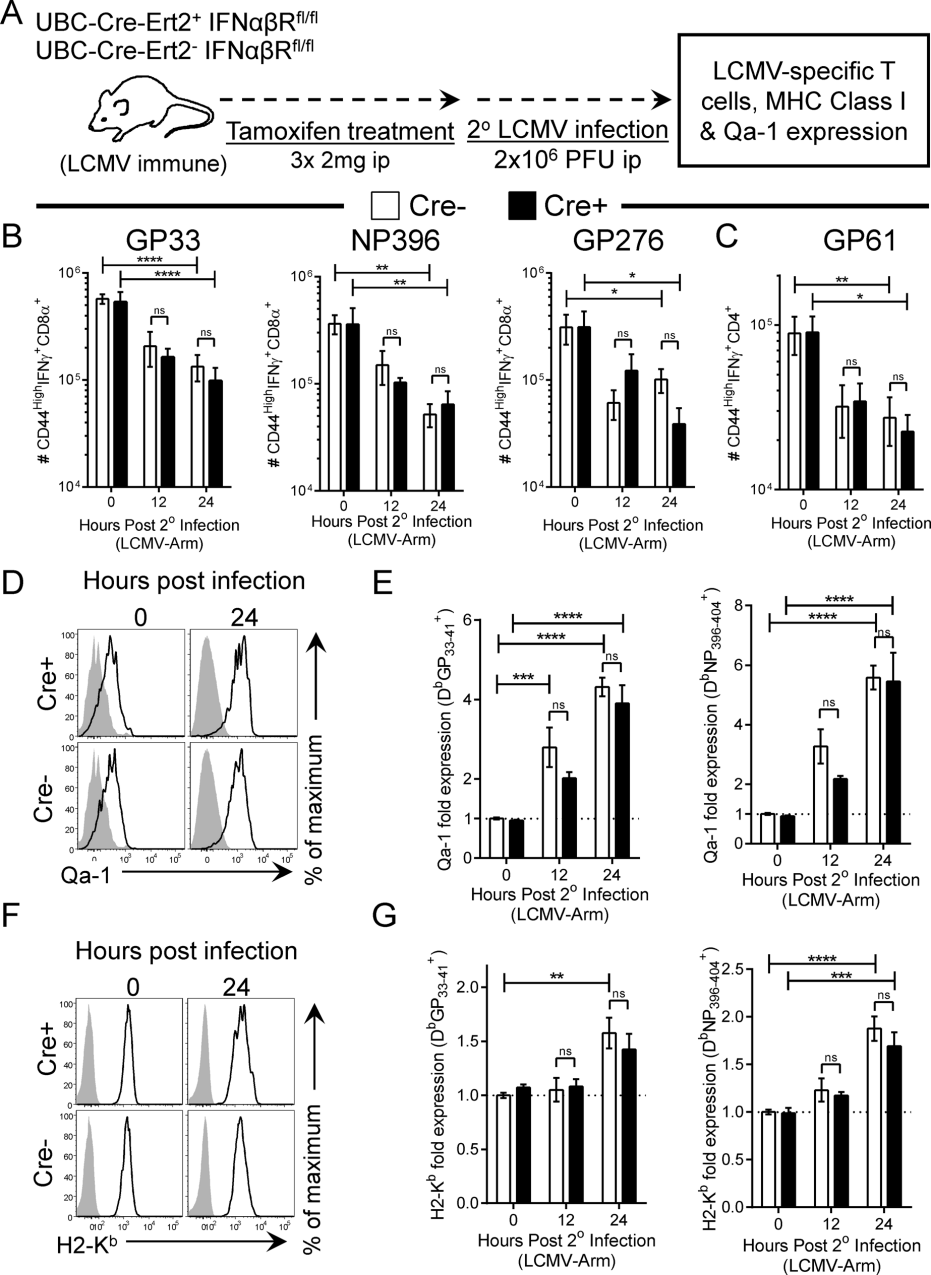
IFNαβR deletion prior to secondary viral infection does not limit LCMV-specific T cell attrition or the expression of MHC Class I & Qa-1 in vivo. (A) LCMV immune UBC-CreERT2^+^ and UBC-Cre-ERT2^-^IFNαβR^fl/fl^ mice were tamoxifen injected and rechallenged with LCMV or given a sham injection. LCMV-specific (B) CD8^+^ and (C) CD4^+^ T cells were quantified via standard intracellular cytokine staining at the indicated times p.i. (D & E) Qa-1 and (F & G) MHC Class I expression was measured upon D^b^GP_33-41_^+^ (left column) and D^b^NP_396-404_^+^ (right column). Shown in panels D & F are representative histograms (gated on D^b^GP_33-41_^+^ CD8^+^ T cells). Data in B, C, E, & G are summations of 2 independent experiments (n = 4–7). Significance was determined via two-way ANOVA with Sidak correction (**** p<0.0001, *** p<0.001, ** p<0.01, * p<0.05).

In the absence of T1IFN signaling, CD8^+^ T cells fail to sufficiently upregulate NK inhibitory receptors, such as MHC Class I and Qa-1, during primary viral infections, and therefore become subject to NK cell mediated lysis, limiting their *in vivo* expansion [20,21]. In addition, T1IFN has been shown to promote the attrition of memory CD8^+^ T cells, in particular, during either viral infection or poly IC injection [26,34]. To determine whether the deletion of IFNαβR from normal memory T cells prior to secondary viral infection would limit T cell attrition and/or abrogate MHC class I or Qa-1 expression, LCMV-immune UBC-Cre-ERT2+ and UBC-Cre-ERT2- IFNαβR^fl/fl^ mice were treated with tamoxifen, then rechallenged with LCMV-Arm, as outlined in Fig 4A, or given a sham infection. Splenocytes were harvested at the indicated hours p.i., and LCMV specific CD8^+^ and CD4^+^ T cells were identified and enumerated using standard intracellular cytokine staining (Fig 4B and 4C) or Class I tetramers (Fig 4D–4G). As we have previously observed [35], immediately following viral infection the absolute number of LCMV specific CD8^+^ and CD4^+^ memory T cells within the spleen rapidly declines; after 24 hours, virus specific T cells were reduced approximately 4- to 10-fold in Cre- mice (Fig 4B and 4C, white bars). Contrary to the predictions based on published studies using conventional KO mice, cells lacking the IFNαβR (Cre+/black bars) contracted to very similar extents, indicating that memory T cell attrition does not depend upon T1IFN signaling. We next assessed whether the absence of IFNαβR limited MHC Class I and/or Qa-1 expression upon virus specific CD8^+^ T cells during the first 24 hours of secondary viral infection. Resting IFNαβR sufficient (Cre-) and IFNαβR deficient (Cre+) D^b^GP_33-41_^+^ or D^b^NP_396-404_^+^ specific memory CD8^+^ T cells expressed limited Qa-1 (Fig 4D and 4E, 0 time point). Immediately following viral infection, at 12 and 24 hours p.i., Qa-1 was significantly upregulated upon both LCMV specific memory CD8^+^ T cell populations, and, additionally, Qa-1 expression was not reliant upon IFNαβR signaling; both Cre- (white bars) and Cre+ (black bars) CD8^+^ T cells expressed similar levels of Qa-1 throughout the first 24 hours of infection (Fig 4D & 4E). Similarly, MHC Class I (H2-K^b^) expression was also comparably increased upon both Cre+ and Cre- D^b^GP_33-41_^+^ and D^b^_NP396-404_^+^ specific memory CD8^+^ T cells in the hours following viral infection (Fig 4F & 4G). Therefore, deletion of IFNαβR from previously-normal virus specific memory T cells does not spare them from attrition during secondary viral infection, and the cells remain capable of upregulating Qa-1 and MHC Class I.

### Secondary expansion, long term maintenance, and qualitative changes of memory CD8+ and CD4+ T cells are not reliant upon T1IFN signaling

**Fig 5:**
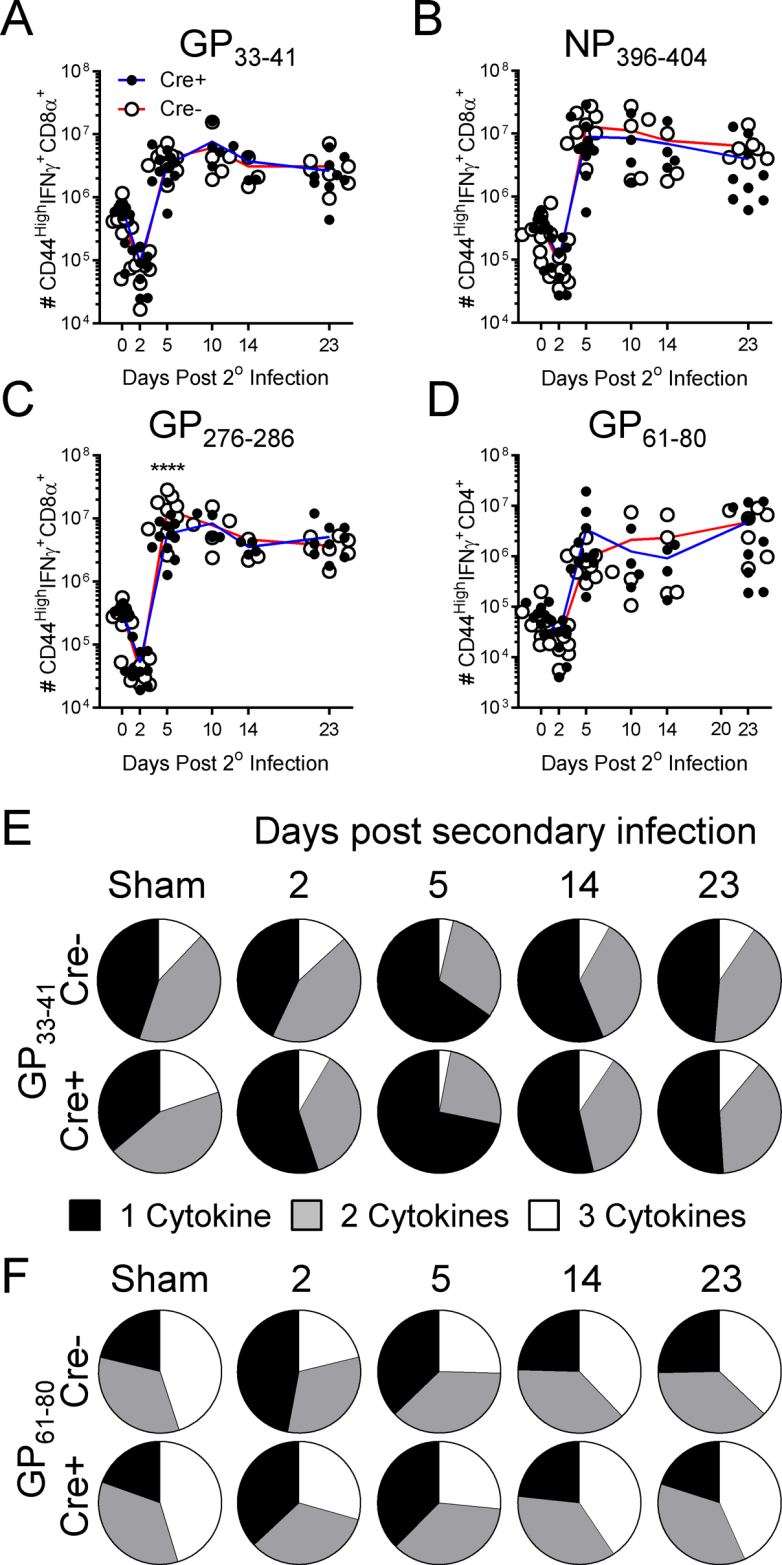
Secondary expansion, long term maintenance, and qualitative changes of memory CD8+ and CD4+ T cells are not reliant upon T1IFN signaling. LCMV-immune UBC-Cre-ERT2+ and UBC-Cre-ERT2-IFNαβR^fl/fl^ mice were injected with tamoxifen and later rechallenged with LCMV. (A-C) CD8^+^ and (D) CD4^+^ T cells specific for the indicated LCMV immunodominant epitope were identified and quantified within the spleen at the indicated times post infection, using standard intracellular cytokine staining. Data are a summation of five independent experiments; each dot represents an individual mouse (at each time point, n = 4–11 per group). (E & F) The ability of CD8^+^ (E) and CD4^+^ (F) memory T cells to produce IFNγ, TNF, and IL-2 was examined using a standard ICCS assay at days 2, 5, 14, and 23 following secondary challenge. The loss of multifunctionality in memory cells soon after secondary infection, and its gradual restoration, are shown by the relative proportion, at each time point, of cells that are capable of producing one, two or three cytokines (black, grey and white segments, respectively).

Previous reports, using conventional IFNαβR knockout mice or adoptively-transferred IFNαβR-deficient CD8^+^ T cells, have documented severe deficits in the ability of IFNαβR deficient memory CD8^+^ T cells to undergo secondary expansion [20,22], and this was attributed to their failure to escape NK cell mediated attenuation [20]. Since we observed no deficit in MHC Class I or Qa-1 expression on memory CD8^+^ T cells following secondary viral infection in our inducible model, we next evaluated the secondary expansion of these IFNαβR-deficient cells, determined their capacity to establish secondary memory T cells. LCMV immune Cre^+^ and Cre^-^ IFNαβR^f/f^ were generated, treated with tamoxifen, and exposed to secondary LCMV infection as in Fig 4A, and five timepoints thereafter, immunodominant virus-specific CD8^+^ (Fig 5A–5C) and CD4^+^ (Fig 5D) T cells were enumerated. At each time point, mice were sacrificed (4–11 per group per time point), and virus-specific cells were enumerated. No difference in attrition (day 2 p.i.), secondary expansion (days 5 & 10 p.i.) or the establishment of long-term 2° memory (days 14 & 23 p.i.) was observed, for either CD8^+^ or CD4^+^ memory T cells (Fig 5). Although we observed no deficit in the total numbers of virus-specific T cells across the course of the secondary response, we reasoned that the quality of the virus specific T cells might nevertheless have been impacted in the absence of T1IFN signaling. We therefore assessed the proportion of LCMV-specific CD8^+^ and CD4^+^ T cells capable of producing IFNγ, TNF, and/or IL-2 following in vitro peptide stimulation. We have previously shown that virus specific memory T cells become predominantly monofunctional (capable of producing only one cytokine) soon after secondary viral infection, and their multifunctionality (i.e., their ability to rapidly produce 2–3 cytokines) is restored over the following 2–3 weeks [35,36]. As expected, these findings are recapitulated, for both CD8^+^ and CD4^+^ T cells, in the genetically-intact mice in this study (Cre^-^ mice in Fig 5E and 5F). The increased monofunctionality at days 2 & 5 p.i. is demonstrated by the increasing size of the black segment, and the concomitant shrinkage of the grey and white areas; and the opposite occurs at days 14 and 23 p.i., indicating the gradual reappearance of multifunctionality. More importantly, these qualitative changes were almost identical, both in kinetics and in extent, in the absence of functional IFNαβR (Cre^+^ mice in Fig 5E and 5F). Thus, the overall kinetics and relative quality of memory T cell responses to secondary viral reactivation appear to be independent of T1IFN signaling.

### Reporter-negative (IFNαβR-intact) CD8+ or CD4+ memory T cells do not preferentially expand during the recall response

**Fig 6:**
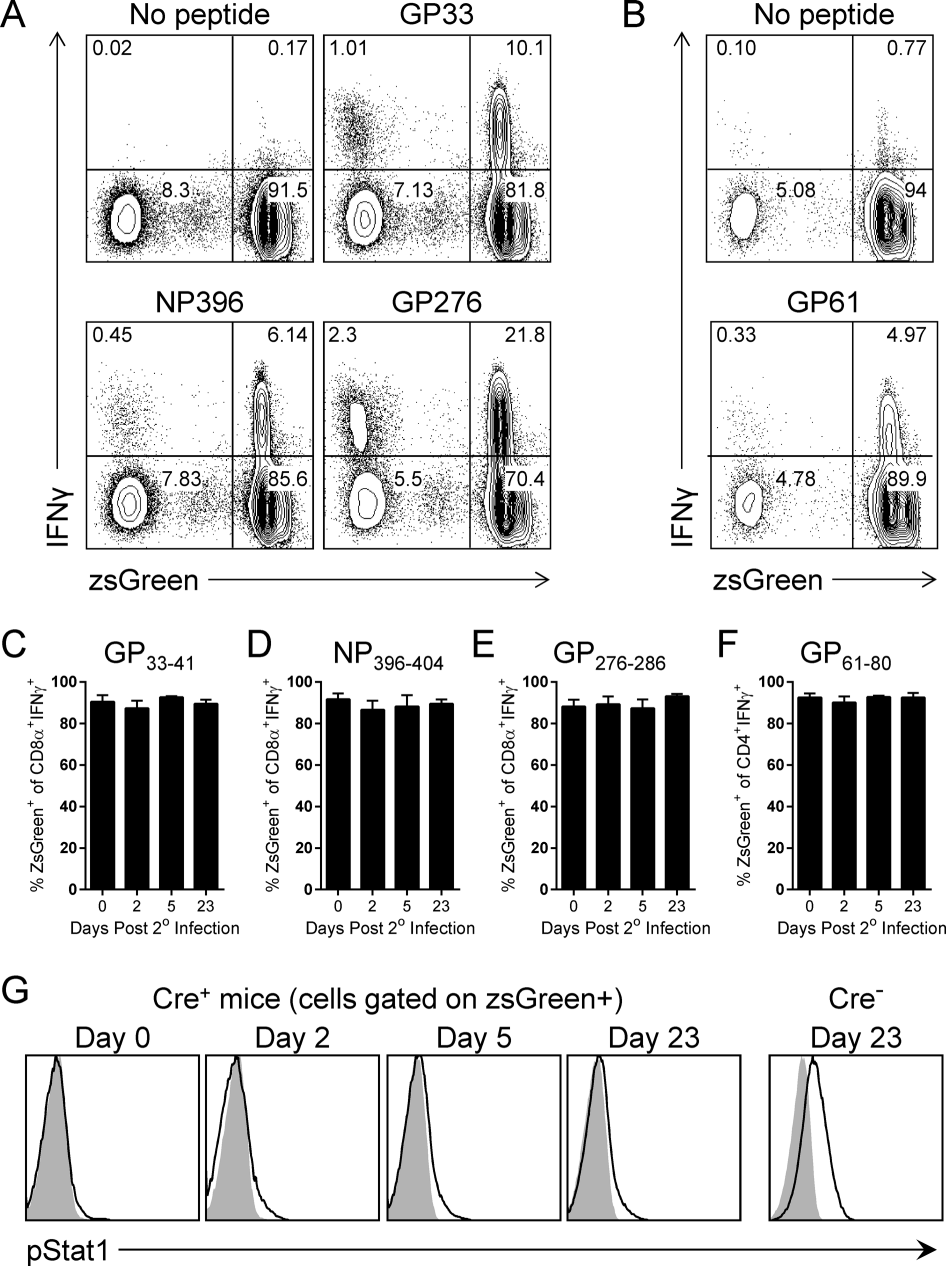
Reporter-negative (IFNαβR-intact) CD8+ or CD4+ memory T cells do not preferentially expand during the recall response. As described in Fig 3A, LCMV-immune Cre reporter (zsGreen) mice were generated, treated with tamoxifen, and subjected to secondary LCMV infection. Mice were sacrificed, and virus-specific CD8^+^ and CD4^+^ T cells were enumerated using intracellular cytokine staining, and evaluated for zsGreen expression, days 0, 2, 5 & 23 following the secondary infection. Representative epitope-specific responses in individual mice are shown. Cells were harvested at day 23 following secondary infection, and are gated on (A) CD8^+^ T cells or (B) CD4^+^ T cells. Numbers represent the proportion of the gated cells in each quadrant. (C-F) Cumulative data showing the proportion of zsGreen-positive cells at the indicated time points over the course of the recall response, in each of the four epitope-specific T cell populations. (G) At the indicated time points, splenocytes from Cre^+^ mice were exposed in vitro to IFNβ, and levels of pSTAT expression were evaluated. Representative plots are shown, gated on CD8 and zsGreen. The rightmost panel is a positive control, showing the responsiveness to IFNβ of day 23 post-secondary CD8^+^ T cells from Cre^-^ animals. Grey histograms = isotype control antibody.

The above data show conclusively that, over the entire course of the recall response, the numbers and quality of virus-specific T cells present in Cre^+^ and Cre^-^ mice are almost identical. However, it is formally possible that this outcome could have resulted from the selective expansion, in Cre^+^ mice, of the small proportion of virus-specific memory T cells (~5%, see Fig 3C and 3D) that had escaped tamoxifen-driven Cre activity. In this scenario, these putative IFNαβR-expressing cells in Cre^+^ mice would, over time, come to predominate the pool of the virus-specific T cells. To assess this, we again exploited Cre reporter (zsGreen) mice. LCMV-immune Cre^+^ IFNαβR^f/f^ mice were generated, treated with tamoxifen, and subjected to secondary LCMV infection (as described in Fig 3A) and, at the indicated time points p.i., mice were sacrificed and zsGreen-positive and -negative virus-specific CD8^+^ and CD4^+^ T cells were enumerated using intracellular cytokine staining (Fig 6). Representative contour plots from day 23 p.i. data for CD8^+^ and for CD4^+^ T cells are shown (Fig 6A and 6B respectively). For all epitopes, >90% of the responding (i.e., IFNγ^+^) cells were zsGreen+, suggesting that zsGreen-negative cells (which should presumably express functional IFNαβR) did not have a marked selective advantage over the course of the recall response. This conclusion is strengthened by cumulative data, from multiple mice at various time points throughout secondary infection (C-F); all of the responding CD8^+^ and CD4^+^ populations maintained a high, and stable, proportion of zsGreen-expressing cells. Finally, we further considered the possibility that, in individual T cells, Cre-reporter activation may not always be accompanied by the deletion of IFNαβR. In that case, zsGreen expression would not invariably reflect the IFNαβR status of the T cell, which would complicate the interpretation of the data in panels A-F. However, if this were the case, and if IFNαβR provided a selective advantage, one would expect to see increasing numbers of zsGreen-positive T cells that remained functionally receptive to T1IFNs. We evaluated this over the course of the recall response (Fig 6G), and saw no significant increase in the IFNβ-responsiveness of zsGreen^+^ T cells. Together, the data in Fig 5 and Fig 6 provide strong support for our assertion that T1IFN signaling plays little or no role in supporting the attrition, secondary expansion, or stable secondary memory formation of memory T cells.

### Reduced T1IFN-inducible gene expression does not limit viral control within the spleen or liver during secondary viral infection

**Fig 7:**
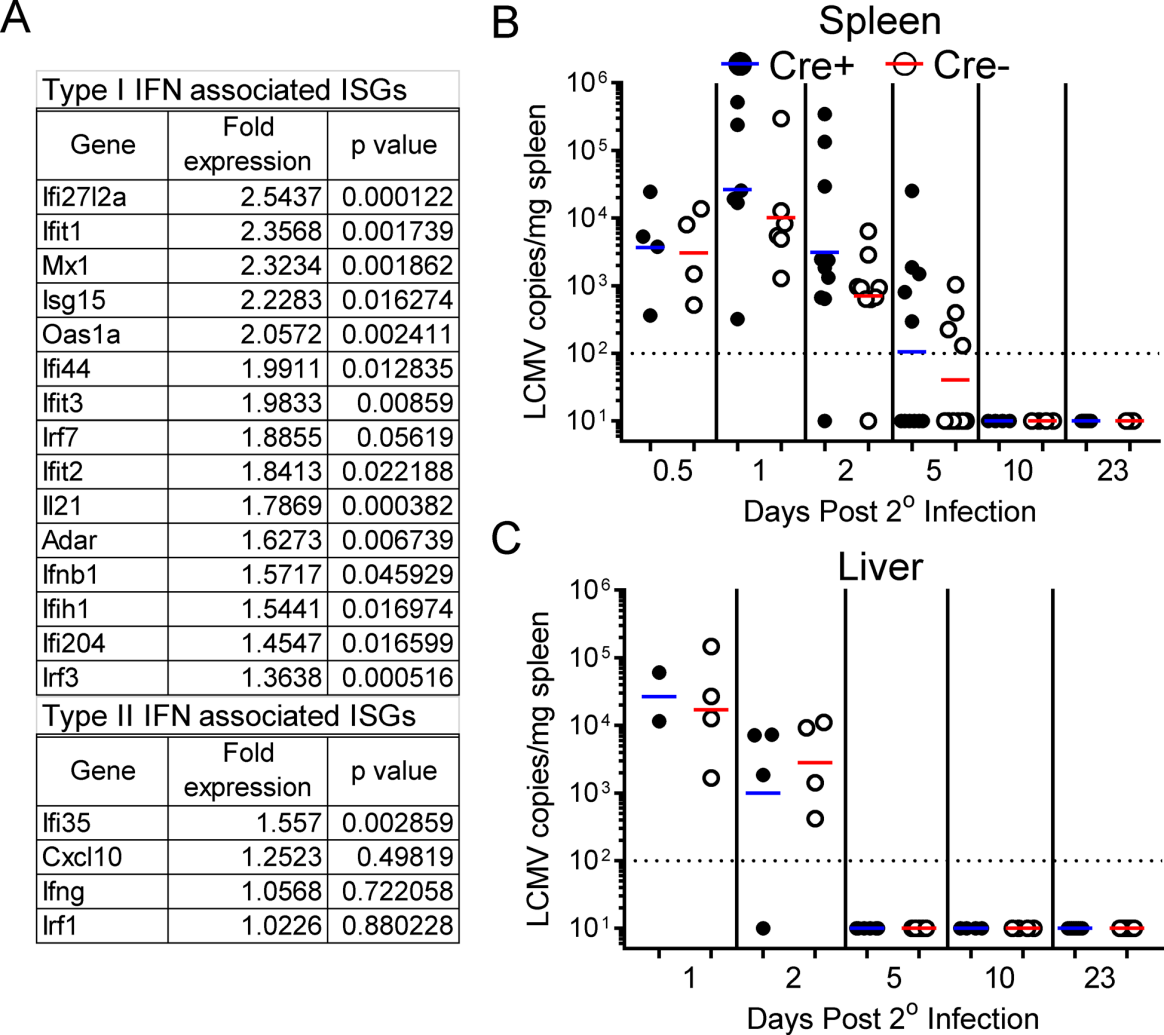
Reduced T1IFN-inducible gene expression does not limit viral control within the spleen or liver during secondary viral infection. (A) Type I and II ISG expression at 1 day post-secondary LCMV infection was assessed via PCR array, in LCMV-immune tamoxifen-treated Cre^+^ and Cre^-^ IFNαβR^f/f^ mice (Cre^+^ n = 5, Cre^-^ n = 6). Relative expression (Cre^-^ /Cre^+^) is shown for each ISG, together with statistical significance. (B & C) LCMV RNA was quantified by qPCR in the spleens (B) and livers (C) of Cre+ and Cre-IFNαβR^fl/fl^ mice at the indicated times following secondary challenge. Data are summations of two (A & C) and five (B) independent experiments. Each dot in panel B & C represents an individual mouse.

Lastly, we determined the ability of tamoxifen-treated Cre^+^ mice to control the secondary LCMV challenge. Published data suggest that their ability to do so may be compromised, for at least two reasons. First, T1IFNs are a key component of the innate response, and act by triggering the expression of a large number of interferon-stimulated genes (ISGs), many of which are antiviral. However, in our model, tamoxifen-induced Cre activation of LCMV-immune mice leads to widespread ablation of IFNαβR (Fig 3), which should limit the capacity of these innate cytokines to stimulate ISG expression. Second, the IFNαβR-deficient memory cells–although exhibiting normal kinetics of attrition, expansion, and secondary memory formation (Fig 5)–may themselves be dysfunctional: previous work has indicated that adoptively-transferred memory CD8^+^ T cells derived from naïve IFNαβR-deficient TCR transgenic cells were unable to control secondary LCMV infection [22]. We first compared the extent of ISG expression at day 1 following secondary infection of tamoxifen-treated LCMV-immune Cre^+^ and Cre^-^ IFNαβR^f/f^ mice (Fig 7A). Type I dependent ISGs known to be capable of directly limiting viral replication [3,37] were significantly elevated in the spleens of Cre^-^ mice, relative to Cre+ mice, with very high levels of statistical significance; type II interferon associated ISG expression–with the exception of Ifi35 –were similar in Cre+ and Cre- infected mice. To determine the impact of IFNαβR ablation on viral control, we used qPCR to quantitate LCMV viral RNA in the spleens (Fig 7B) and livers (Fig 7C) of LCMV infected Cre+ and Cre- mice at the indicated time points following secondary challenge. Regardless of IFNαβR status, LCMV vRNA was cleared from both organs at near-equivalent rates. Strikingly, no statistically-significant differences were observed even at early (0.5 and 1 day p.i.) times, when T1IFNs would be expected to exert the greatest anti-viral effect. Moreover, complete viral clearance appeared to be achieved in the Cre^+^ mice; no viral recrudescence was observed as late as >3 weeks after secondary challenge. Thus, antigen-specific CD8^+^ and CD4^+^ T cells can mount protective antiviral recall responses that are independent of IFNα/β signaling; this is consistent with their similar kinetics of expansion (Fig 5) and their ability to escape NK cell mediated lysis through the upregulation of MHC class I and Qa-1 (Fig 4).

## Discussion

T1IFN is a well-established mediator of anti-viral immunity during primary infection, directly limiting viral replication by upregulating ISGs in many somatic cells, and activating and regulating the innate and adaptive arms of the immune system [1–5]. The role of these cytokines during the recall response is far less well-characterized, but extant studies have proposed that T1IFNs also influence the responses of memory T cells [reviewed, 38]. However, those studies relied on memory cells that had been generated during a primary infection of T1IFN-deficient mice, or from receptor-deficient precursor T cells, and one can conceive of at least two ways in which these techniques might have affected the memory cells’ development: first, directly–the cells will not receive direct T1IFN signals; and, second, indirectly–virus and antigen load will be abnormally high, and prolonged, during primary infection in T1IFN-deficient animals, potentially modulating the maturation of virus-specific cells. Herein, we have employed an inducible deletion model in which both of these potential confounders are nullified. The primary viral infection, and the corresponding immune response, are entirely normal; only after memory cells are established (>6 weeks following the primary infection) is IFNαβR inducibly deleted.

During secondary LCMV infection, IFNα and IFNγ are transiently produced during the first 24 hours of infection (Fig 1). We [35,39] and others [40] have previously reported the transient burst of IFNγ by CD8^+^ T cells in response to homologous and heterologous secondary viral infection; the focus of the present study was on T1IFN. Relative to primary LCMV infection [28], peak levels of IFNα during secondary LCMV infection are substantially reduced but remain sufficient to drive the significant upregulation of a number of T1IFN dependent ISGs in the spleen at 12 hours p.i. (Fig 1C); these findings validated our investigation of the impact of these cytokines on memory T cells. We developed a tamoxifen-sensitive inducible system to delete IFNαβR from all cells and demonstrated that, during primary viral infection (Fig 2), T cells in mice that had been pre-treated with tamoxifen failed to appropriately expand, and the mice were unable to control the viral infection. These data largely recapitulated published studies using either conventional IFNαβR knockout mice, or recipients of transferred IFNαβR knockout CD8^+^ T cells [6,7,16,17,20,21], providing additional validation for our model. The profound biological impact of tamoxifen administration also indicated not only that *in vivo* Cre-mediated deletion of floxed IFNαβR DNA must be efficient, but also that it must have led to the rapid and widespread loss of any existing functional IFNαβR. These observations were confirmed by our analyses of tamoxifen-treated LCMV-immune mice (Fig 3), in which IFNαβR deletion was near-complete in genomic DNA extracted from all tested tissues, and in which Cre activity was demonstrable in the vast majority of CD8^+^ and CD4^+^ T cells as shown by the use of a zsGreen reporter line. Most importantly, we confirmed that the genetic disruption of IFNαβR was accompanied by the rapid cessation of receptor function; Cre reporter^+^ CD8^+^ T cells were unable to phosphorylate Stat1 in response to *in vitro* IFNβ stimulation (Fig 3E).

Several studies have shown that T1IFNs can cause a loss of antigen-specific and nonspecific memory (and to a lesser extent naïve) CD8^+^ T cells during viral infection *in vivo*, in a process termed attrition [25,26,34]. We have previously confirmed that attrition occurs [35], and Fig 4B shows that a large proportion of LCMV-specific memory CD8^+^ T cells are, indeed, lost from the spleen within the first 24 hours of secondary LCMV infection. However, and to our surprise, the ablation of IFNαβR from normal memory T cells prior to the secondary viral infection did not prevent this attrition, indicating that an alternative pathway(s) must be contribute to the process; we speculate that this may be driven by CD8^+^ T cell derived IFNγ, which is produced in the hours immediately following secondary viral infection (Fig 1) [35]. In addition to being involved in attrition, direct IFNα/β signaling leads to the specific upregulation of MHC Class I and Qa-1 on CD8^+^ T cells during *in vitro* stimulation and/or primary viral infection *in vivo*, and the failure to appropriately upregulate these and other inhibitory molecules allows the cells to be attacked by NK cells, limiting the expansion of the virus-specific CD8^+^ T cells [21]. Qa-1, the mouse analog of human HLA-E, acts as an external readout for intracellular MHC class I processing [41], binding to the inhibitory receptor NKG2a [42] and suppressing NK cell activation [43]. LCMV-specific IFNαβR-deficient memory CD8^+^ T cells upregulated both MHC class I and Qa-1 *in vivo* (Fig 4D–4G), suggesting that NK-mediated killing may not contribute to the cells’ rapid attrition; moreover, this upregulation occurred in both Cre^-^ and Cre^+^ mice, indicating that the expression of MHC Class I and Qa-1 by memory CD8^+^ T cells also can occur independently of IFNα/β signaling. In summary, multiple studies have shown that both T cell attrition and the upregulation of MHC class I & Qa-1 can be triggered by T1IFN, but our data indicate that there is functional redundancy in memory T cells which allows these changes to take place even when IFNαβR signaling is abrogated. We infer, from the existence of this redundancy, that these biological events–attrition, and upregulation of protective molecules–may play a key role in ensuring a balanced and appropriate memory T cell response.

More detailed analyses of the kinetics of the secondary T cell responses (Fig 5) confirmed that attrition (day 2) was unaffected by ablation of IFNαβR, and also showed that the subsequent expansion of the T cells, their ability to produce multiple cytokines, and their entry into secondary memory, were both entirely normal in IFNαβR-ablated animals. This is in stark contrast to the primary immune response, in which T1IFN signaling plays a key supporting role in T cell expansion, and provides a powerful selective pressure favoring effector CD8^+^ T cells that bear the receptor [16,17,20,21]. We considered the possibility that such pressures may have promoted the selective expansion of the few memory cells that had evaded tamoxifen-induced Cre activation prior to secondary LCMV infection; theoretically, this could have provided an alternative explanation for why IFNαβR-sufficient (Cre^-^) and IFNαβR-ablated (Cre^+^) animals had near-identical numbers of virus-specific cells over the course of the recall response. However, the proportion of zsGreen-positive cells remained stable over time (Fig 6), furthering reinforcing our conclusion that all phases of the CD8^+^ and CD4^+^ virus specific recall response are independent of T1IFN signaling. These findings are discordant with published data using conventional knockout mice, in which memory cells were reliant on T1IFN signaling. We propose that those memory cells, which had developed in an environment devoid of T1IFN signaling, were intrinsically abnormal; indeed, it is possible that even their naïve precursors were, in some unknown way, defective. Finally, we evaluated the importance of T1IFN signaling during secondary virus infection. Compared to naïve mice, LCMV-immune mice rapidly control viral replication, and significant differences in viral RNA are detectable within as few as 6 hours of infection [35]. T1IFNs are rapidly expressed during the recall response (Fig 1) [28], so it was possible that these cytokines might have contributed to this antiviral effect. Conceivably, they could have done so in two ways, by (i) upregulating ISG expression in somatic cells and/or (ii) promoting the effector functions of virus-specific T cells, a suggestion supported by previous work showing that the antiviral effector function of IFNαβR-deficient memory CD8^+^ T cells is defective *in vivo* [22]. Therefore, we questioned whether the de novo ablation of IFNα/β signaling prior to secondary infection would inhibit the control of LCMV in the spleen and the liver. We found that the inducible deletion of IFNαβR had no significant impact on the containment and ultimate clearance of a secondary viral infection (Fig 7B and 7C). Additionally, viral recrudescence was undetectable as late as 23 days p.i. Thus, we contend that–contrary to current thinking–T1IFN signaling is not required for the secondary response to viral infection, neither regulating the attrition, expansion, and secondary memory formation of memory CD8^+^ and CD4^+^ T cells, nor being required for establishing an antiviral state within host parenchymal cells. It is possible that, in the absence of T1IFN signaling, other inflammatory cytokines may contribute to supporting these key biological functions.

Two additional points are worthy of consideration. *First*, it has been known for some time that memory T cells can very rapidly suppress viral replication. Viral RNAs are one of the major instigators of the T1IFN response, and we recently showed that–when compared to naïve mice–a reduction in RNA levels was detectable as early as 6 hours p.i. in LCMV-immune mice, and there was a >100-fold reduction in viral RNA by 12 hours after LCMV challenge [35]. Thus, the recall response very quickly prevents the accumulation of molecules that drive T1IFN production; in that light, it makes good evolutionary sense that the recall response should not rely on T1IFNs, and should be effective in its absence. *Second*, in recent years the concept of “innate memory” has come to the fore. Our findings suggest that T1IFNs are not required for the control or eradication of secondary viral challenge.

How might these observations relate to human immune responses and viral diseases? Genetic deficiencies in IFNγ signaling (and related pathways) are increasingly being identified; perhaps surprisingly, sufferers are not particularly susceptible to most viral infections, instead being vulnerable to infection by mycobacteria [44]. To date, fewer defects in T1IFN responses (and related pathways) have been reported in humans, and we speculate that such deficiencies may be extremely rare because they confer a dramatic evolutionary disadvantage, by rendering the victims open to severe primary viral infections. Nevertheless, there are some examples, in humans, of mutations that disrupt T1IFN responses. Homozygous mutation in IRF7 led to a near-fatal infection by influenza virus [45], while mutations in TLR3 [46] and in several other T1IFN-related genes [47] markedly increase the risk of herpes simplex encephalitis (HSE). This condition can be treated with acyclovir, and, although fatal in some individuals, the majority survive, albeit usually with neurological sequelae. However, and notably, it appears that the survivors are not at high risk for HSE recurrence; we speculate that the therapeutic intervention led to the rapid suppression of the primary infection, allowing these patients to develop memory T cell responses; thereafter, these patients are, in essence, phenotypically similar to our LCMV-immune, tamoxifen-treated Cre^+^ mice; despite being unable to respond to T1IFN signaling, they are protected against secondary exposure to the virus.

In conclusion, we show here that T1IFNs are dispensable during a secondary viral infection. This finding contrasts with published studies, and we suggest that this discrepancy has broad implications. Many–indeed almost all–analyses of how individual genes contribute to memory T cell responses have relied on approaches in which the knockout is either present in the germline (conventional KO), or is conditional (e.g., Cre is expressed from a cell-specific promoter), and, in almost all of those cases, the genetic defect is present prior to the primary infection. Using these approaches to study IFNαβR, defective expansion by memory T cells was observed; it was inferred, therefore, that the memory T cell response–like that of their primary counterparts–must benefit from T1IFN signaling. However, our data strongly suggest that the memory cells upon which those inferences depend are intrinsically abnormal, presumably because they arose and developed in the absence of T1IFN signaling, and, therefore, such memory cells cannot reliably be used to assess how individual genes affect the recall T cell response. Thus, we contend that it is vital that the evaluation of any gene’s contribution to secondary (and subsequent) immune responses should employ an approach that does not compromise the very response from which those memory cells arise.

## Materials and Methods

### Ethics statement

All animal experiments were approved by The Scripps Research Institute (TSRI) Institutional Animal Care and Use Committee and were carried out in accordance with the National Institutes of Health’s *Guide for the Care and Use of Laboratory Animals*.

### Mice, virus, & Cre activation

C57BL/6J mice were purchased from the TSRI rodent breeding colony. Transgenic inducible Cre mice under the control of the human ubiquitin promoter (B6-UBC-Cre-ERT2, JAX 008085) [48] and IFNαβR^fl/fl^ mice [49] were crossed together to generate UBC-Cre-ERT2^+^IFNαβR^fl/fl^ (referred to as Cre^+^IFNαβR^fl/fl^). To generate LCMV-immune mice, Cre^+^IFNαβR^fl/fl^ and control littermate Cre^-^IFNαβR^fl/fl^ mice were challenged with 2x10^5^ PFU LCMV-Arm (Armstrong strain) intraperitoneally (i.p.). >6 weeks following LCMV infection, Cre+ and Cre- mice were injected with 3 successive, daily i.p. doses of 2mg of tamoxifen dissolved in corn oil. At least two weeks were allowed to elapse after tamoxifen injection before the Cre^+^IFNαβR^fl/fl^ and Cre^-^IFNαβR^fl/fl^ mice were rechallenged with LCMV-Arm (2x10^6^ PFU i.p.). For experiments using a Cre reporter, IFNαβR^fl/fl^ mice were crossed and backcrossed with zsGreen Cre-reporter mice (B6.Cg-Gt(ROSA)^26Sortm6(CAG-ZsGreen1)Hze^/J, JAX 007906 [50]) to generate doubly-homozygous IFNαβR^f/f^zsGreen^+/+^ animals. These mice were crossed against Cre^+^IFNαβR^fl/fl^ animals, providing F1 mice that were all IFNαβR^f/f^zsGreen^+/wt^, and were either Cre^+^ or Cre^-^. Two weeks following tamoxifen administration, flow cytometry was used to identify T cells in which Cre activity had occurred, indicated by zsGreen expression. Mice were genotyped with the following primers (5’-3’): UBC-Cre-ERT2 (F) GCC AGC TAA ACA TGC TTC ATC GT & (R) CGC GGC AAC ACC ATT TTT TCT GA, IFNαβR^fl/fl^ (F) AAG CTC CTT GCT GCT ATC TG & (R) CAC ACC AGG CTT CTA ATG TC, and Cre-Reporter (WT F) AAG GGA GCT GCA GTG GAG TA, (WT R) CCG AAA ATC TGT GGG AAG TC, (Tg F) AAC CAG AAG TGG CAC CTG AC, & (Tg R) GGC ATT AAA GCA GCG TAT CC. To assess UBC-Cre-ERT2 mediated deletion of IFNαβR, genomic DNA was extracted from various tissues using Qiagen DNeasy Blood & Tissue Kit, then used as a PCR template with the above IFNαβR^fl/fl^ primer set. These primers flank both loxp sites and exon 10 of IFNαβR, thereby discriminating between WT, floxed, and deleted alleles. Analysis of IFNαβR deletion was routinely carried out for every mouse in this study.

### Measurement of IFNα & IFNγ

LCMV-immune (>6 weeks p.i.) C57BL/6 mice were rechallenged with 2x10^6^ PFU LCMV-Arm ip, and blood was collected at the indicated times following secondary LCMV infection. Plasma was isolated using K3-EDTA coated microvette tubes (Starstedt, Nümbrecht, DEU), aliquoted, and stored at -80°C. Interferon alpha levels within plasma were assessed using Verikine Mouse Interferon Alpha ELISA kit (PBL Assay Science, Piscataway, NJ), and interferon gamma was determined by LEGENDplex Mouse Inflammation panel multiplex (Biolegend, San Diego, CA) according to manufacturer instructions.

### PCR array and quantitative PCR

Interferon stimulatory gene expression within the spleens of wildtype C57BL/6 and Cre+ and Cre-IFNαβR^fl/fl^ mice was quantified via Mouse Interferons & receptors PCR Array (PAMM-064Z, SA Biosciences, Frederick, MD) according to manufacturer instructions. Copies of LCMV vRNA within the spleens of Cre+ and Cre- were assessed using reverse transcriptase real time PCR (qPCR) as previously described [35,51].

### Flow cytometry

Single cell suspensions of splenocytes were obtained from mechanically disrupted and red blood cell lysed spleens. Dead cells were identified and excluded using Zombie NIR Fixable Viability Kit (Biolegend). After Fc-blocking with anti-CD16/32 (BD Biosciences, San Diego, Ca), splenocytes were immunophenotyped with the following fluorescently conjugated antibodies to cell surface markers (Biolegend) CD8α (53–6.7), CD4 (RM4-5), CD44 (1M7), H2Kb (AF6-88.5), & Qa-1 (6A8.6F10.1A6, Milteyi Biotec, Bergisch Gladbach, DEU). MHC-Class I tetramers specific for D^b^GP_33-41_ and D^b^NP_396-404_ were provided by NIH Tetramer Core Facility (Emory University, Atlanta, GA). Appropriately conjugated isotype-control antibodies were used in all experiments. Samples were acquired on a BD Biosciences LSR-II and analyzed using FlowJo (Treestar, Ashland, OR).

### Standard (indirect) intracellular cytokine staining

As previously described [35], 2x10^6^ splenocytes were incubated for 6 hours directly *ex vivo* with GolgiPlug (BD Biosciences) and 1μM of the CD8^+^ epitopes GP_33-41_, NP_396-404_, or GP_276-286_ or 10µM of the CD4^+^ epitope GP_61-80_. Stimulated splenocytes were subsequently washed, stained with viability dye, Fc-blocked, and surface stained with CD4, CD8α, & CD44 as above. After surface staining, splenocytes were washed, fixed with Cytofix/Cytoperm (BD Biosciences) and resuspended in 1X PermWash (BD Biosciences). Intracellular cytokines were identified with fluorescently conjugated antibodies to IFNγ (XMG1.2). Unstimulated (no peptide) cultured splenocytes were used to identify antigen-specific cytokine production.

### In vitro IFNβ stimulation

4x10^6^ splenocytes were stimulated for 25 minutes directly *ex vivo* with or without 10^4^U/ml of recombinant mammalian IFNβ (PBL Assay Science). After stimulation, splenocytes were washed in cold FACS Buffer (1x PBS pH 7.4, 2% FBS, 0.1% Na Azide), fixed with Cytofix/Cytoperm (BD Biosciences), Fc blocked, stained for CD8α as above, permeabilized with Perm Buffer III (BD Biosciences), and phosphorylated Stat1 (pY701) was identified using fluorescently conjugated antibodies (4a, BD Biosciences). The functional deletion of IFNαβR signaling was assessed for all mice presented in this study.

### Statistical analysis

Significant differences were assessed via one or two way ANOVA with Sidak correction where appropriate, and results were considered significant if P<0.05 (Prism 7, Graphpad, San Diego, CA).
